# How do Dutch general practitioners detect and diagnose atrial fibrillation? Results of an online case vignette study

**DOI:** 10.1186/s12875-019-1064-y

**Published:** 2019-12-14

**Authors:** N. (Nicole) Verbiest - van Gurp, D. (Dominique) van Mil, H. A. M. (Henri) van Kesteren, J. A. (André) Knottnerus, H. E. J. H. (Jelle) Stoffers

**Affiliations:** 1 Care and Public Health Research Institute (CAPHRI), Department of Family Medicine, Maastricht University, Peter Debyeplein 1, 6229 HA Maastricht, Limburg The Netherlands; 2 Department of Cardiology, Admiraal de Ruyter Ziekenhuis, ‘s-Gravenpolderseweg 114, 4462 RA Goes, Zeeland The Netherlands

**Keywords:** Atrial fibrillation, Diagnosis, Electrocardiography, Electrocardiography, Ambulatory, Health care surveys, General practice

## Abstract

**Background:**

Detection and treatment of atrial fibrillation (AF) are important given the serious health consequences. AF may be silent or paroxysmal and remain undetected. It is unclear whether general practitioners (GPs) have appropriate equipment and optimally utilise it to detect AF. This case vignette study aimed to describe current practice and to explore possible improvements to optimise AF detection.

**Methods:**

Between June and July 2017, we performed an online case vignette study among Dutch GPs. We aimed at obtaining at least 75 responses to the questionnaire. We collected demographics and asked GPs’ opinion on their knowledge and experience in diagnosing AF. GPs could indicate which diagnostic tools they have for AF. In six case vignettes with varying symptom frequency and physical signs, they could make diagnostic choices. The last questions covered screening and actions after diagnosing AF. We compared the answers to the Dutch guideline for GPs on AF.

**Results:**

Seventy-six GPs completed the questionnaire. Seventy-four GPs (97%) thought they have enough knowledge and 72 (95%) enough experience to diagnose AF. Seventy-four GPs (97%) could order or perform ECGs without the interference of a cardiologist. In case of frequent symptoms of AF, 36–40% would choose short-term (i.e. 24–48 h) and 11–19% long-term (i.e. 7 days, 14 days or 1 month) monitoring. In case of non-frequent symptoms, 29–31% would choose short-term and 21–30% long-term monitoring. If opportunistic screening in primary care proves to be effective, 83% (58/70) will support it.

**Conclusions:**

Responding GPs report to have adequate equipment, knowledge, and experience to detect and diagnose AF. Almost all participants can order ECGs. Reported monitoring duration was shorter than recommended by the guideline. AF detection could improve by increasing the monitoring duration.

## Background

Atrial fibrillation (AF) can have serious health consequences such as stroke and heart failure. Adequate antithrombotic treatment reduces the risk of stroke by 60% [1, 2]. Unfortunately, AF often remains undetected and untreated, because it can be asymptomatic or paroxysmal. Many studies involving screening and new devices aimed to find ways to increase the AF detection rate [3–8]. A current example of such a study in Dutch primary care is D_2_AF (Detecting and Diagnosing Atrial Fibrillation), a multicentre cluster randomised controlled trial with nested diagnostic studies [9]. The intervention practices of D_2_AF perform opportunistic screening for AF, and the control practices provide usual care.

Innovations such as screening are not the only way to increase AF detection rate; this might also be accomplished by optimising current practice. However, it is unclear how general practitioners (GPs) currently detect and diagnose AF. In the optimal situation GPs would have knowledge and experience regarding AF, adhere to the guideline and have access to diagnostic devices, i.e. 12-lead ECG and preferably also an ambulatory device.

We therefore undertook a survey to explore whether GPs have appropriate equipment and optimally exploit their diagnostic tools for AF detection. This study aimed to describe current practice to see if improvement is possible, in order to optimise the detection of AF.

## Methods

### Study design and setting

For this case vignette study, using six case vignettes with varying characteristics related to AF, we laid our focus on achieving a representative sample of GPs. We performed a sample size calculation to determine the number of responses needed. A sample size of 75 had an acceptable margin of error of 0.11 from the 95% CI in a conservative calculation based on a proportion of 0.5, i.e. the width of the 95%CI does not exceed 0.22. For a proportion of 0.5 this means that the lower limit of the 95%CI is equal to or higher than 0.5–0.11 = 0.39 and the upper limit is equal to or lower than 0.5 + 0.11 = 0.61.

In June and July 2017, we sent our survey to a surplus of GPs (*n* = 385), accounting for the expected low response [10, 11]. This was a random selection of e-mail addresses of GPs from the database of the Department of Family Medicine of Maastricht University, covering the south-eastern part of the Netherlands. To improve the geographical spread, we also used GPs who had participated in the control arm of the nationwide D_2_AF study (*n* = 25) [9]. We excluded GP trainees, current participants in the control arm of the D_2_AF study and all participants in the intervention arm. We sent one general reminder to both responders and non-responders, and a maximum of five reminders to non-responding D_2_AF GPs. No further invitations were sent after the required sample had been achieved. We offered participants a 10-euro gift card.

The Medical Ethics Review Committee of Maastricht University Medical Centre waived formal review because the Medical Research Involving Human Subjects Act (WMO) does not apply.

### Online questionnaire

The questionnaire was adapted from a previous version for cardiologists to fit the situation of GPs [12]. For example, we removed ‘implanted devices’ from the answering options. Questions were multiple choice with room for comments, the language was Dutch. Two GPs and the communication expert of the department of general practice tested the pre-final version. We used Formdesk to present the questionnaire online.

The questionnaire consisted of several parts. Firstly, we inquired after the demographics of respondents and their practice. Subsequently, we asked their opinion on their knowledge and experience in diagnosing AF on a five-point Likert scale. After that, they could indicate which diagnostic devices they have and use to diagnose AF. This was followed by questions on six case vignettes with varying characteristics related to AF (risk factors, signs and symptoms and symptom frequency), as shown in Table 1. These key elements cover the situations in which a GP could be inclined to start a diagnostic process for AF and in which the GP had different diagnostic options according to the guideline [13]. The vignettes described these elements pointwise. The survey concluded with questions on screening and actions after diagnosing AF.

**Table 1:** Description of six case vignettes on AF used in the online questionnaire

	A	B	C	D	E	F
Risk factors for AF (CHA_2_DS_2_-VASc^a^)	X					
No symptoms^b^ of AF	X	X				
Non-frequent symptoms of AF (< 1/24 h)			X	X		
Frequent symptoms of AF (≥1/24 h)					X	X
Signs of AF during physical examination^c^		X		X		X

^a^Congestive heart failure, hypertension, age of 65–74 or > 74, diabetes, stroke, TIA, thromboembolism, vascular disease, female sex

^b^Dyspnoea, exercise intolerance, chest pain, palpitations, dizziness and/or syncope

^c^Irregular pulse, pulse deficit or a varying loudness of the first heart sound

We divided the questions on the case vignettes into two sets. In each case, we first asked whether the GP would start a diagnostic process to detect AF, and if so, with what technique. In the second set of questions, the cases in which the GP would start a diagnostic process with a 12-lead ECG were presented again. We asked if he or she would continue the diagnostic process if the results were negative, and if so, with what technique. If the GP chose Holter or event recording, then he or she had to indicate the monitoring duration.

### Data analysis

We used IBM SPSS Statistics 25 for descriptive statistics and analysis. We performed an independent samples T-test to investigate if the experience of GPs in years is related to whether they consider treating patients themselves. We used McNemar’s test to investigate the association between symptom frequency and monitoring duration. As we used two sets of questions in which respondents could choose to apply monitoring, we combined both sets of answers to evaluate the total number of respondents who would apply monitoring. We dichotomised monitoring duration of both Holter and event recording into short-term (i.e. 24 and 48 h) and long-term (i.e. 7 days, 14 days and 1 month) monitoring. Often Holter is short-term and event recording long-term monitoring, but not necessarily.

Free comments were categorised by theme. We compared the answers of GPs with the guideline of the Dutch College of General Practitioners [13]. Missing values were assumed to be missing at random.

## Results

### Study population

**Fig. 1:**
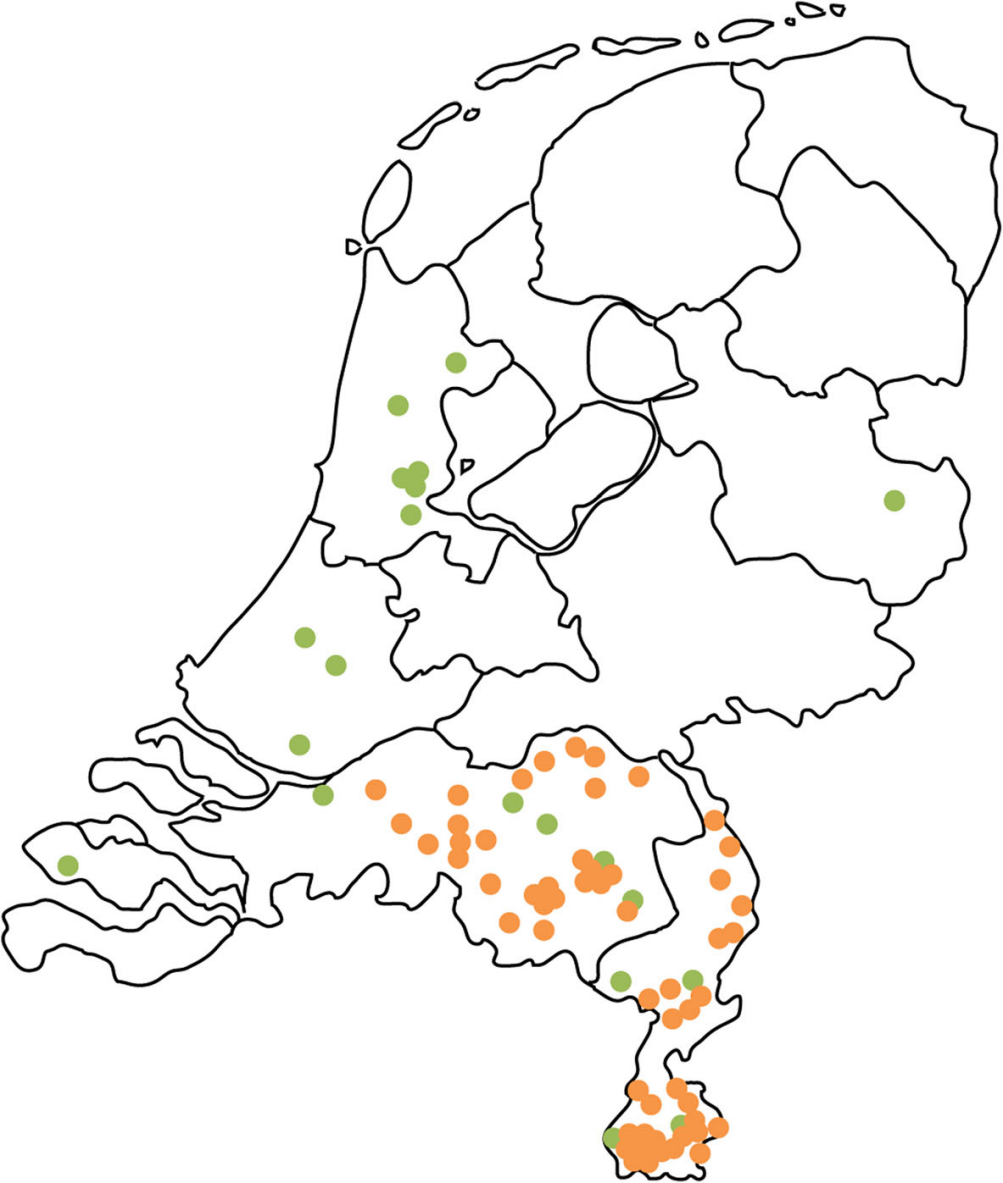
Geographic distribution of responding D_2_AF GPs (*n* = 20, green) and non-D_2_AF GPs (*n* = 56, orange)

We terminated data collection after 76 responses. Respondents’ characteristics are shown in Table 2 and their geographic distribution in Fig. 1. D_2_AF GPs were older than the other GPs (mean age 54.8 vs. 49.2 years, *p* = 0.023), but did not differ in other characteristics.

**Table 2:** Characteristics of responding GPs and their practice

Characteristic	n = 76
Respondents	
Male, n (%)	47 (61.8)
Age in years, mean (range)^a^	50.7 (30–66)
Years of experience, mean (range) ^a^	19.3 (3–39)
Practices	
Number of GPs, mean (range) ^b^	2.99 (1–8)
Number of patients, mean (range)	4496 (1300–11,000)

^a^One GP did not fill in the questions for age and years of experience

^b^Three GPs did not fill in the question on ‘number of GPs’

### Diagnostic equipment

**Fig. 2:**
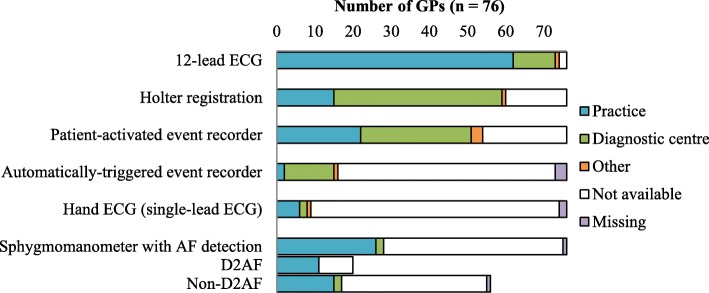
Availability and location of diagnostic devices in AF detection for the GP. Other consisted of pulse palpation, auscultation and determination of the presence of pulse deficit. The availability of the sphygmomanometer is split up for D_2_AF and non-D_2_AF GPs, as the former got a sphygmomanometer with AF detection as a gift for participation in the D_2_AF study

Ninety-seven percent of responding GPs (74/76) felt that they have enough knowledge and 95% (72/76) judged they have enough experience to diagnose AF. GPs have a wide variety of diagnostic techniques at their disposal (see Fig. 2 for details). Ninety-seven percent of GPs (74/76) could order ECGs without the interference of a cardiologist. Eighty-four percent of them (62/74) had an ECG device in-house.

Techniques GPs actually used to diagnose AF were ECGs (72/76, 95%), Holter registrations (37/76, 49%), patient-activated event recorders (33/76, 43%), automatically-triggered event recorders (1/73, 1%), hand-ECGs (7/74, 9%) and sphygmomanometers with AF-detection algorithm (15/75, 20%). Due to missing answers in the questions on the three last devices, the denominator is below 76. Almost all GPs with access to a 12-lead ECG device use it to diagnose AF, whereas approximately half of the GPs with access to monitoring devices like Holters and event recorders seem to use those techniques.

### Diagnostic process

**Fig. 3:**
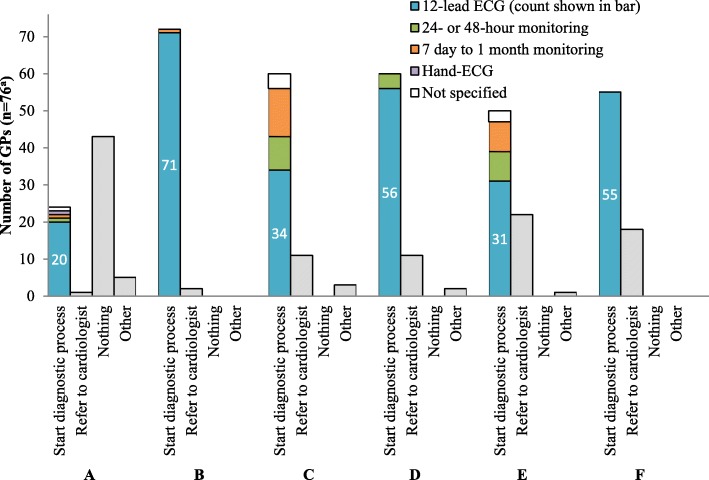
Initial action of GPs per case vignette. a Three GPs did not answer the question for vignette A, D, E and F (*n* = 73), and two GPs did not answer the question for vignette B and C (*n* = 74)

In all vignettes, except vignette A (a patient without signs or symptoms indicative of AF), all GPs would undertake action, either by starting the diagnostic process, referral or something else, as shown in Fig. 3. In vignette A, 33% of GPs (24/73) would start the diagnostic process, and 59% (43/73) would do nothing. In all cases, the majority preferred starting the diagnostic process above direct referral to the cardiologist. Most GPs started with a 12-lead ECG. One GP indicated to consider a single lead ECG as a solitary diagnostic tool for frail homebound elderly.

**Fig. 4:**
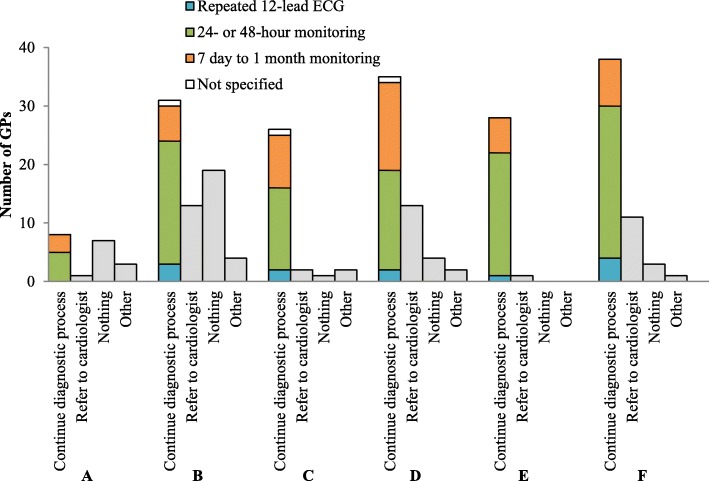
Subsequent action per case vignette of GPs after a negative initial 12-lead ECG. See numbers of GPs whose initial action was to start with a 12-lead ECG in the blue bar of Fig. 3. One GP did not answer the question for vignette A (*n* = 19), four GPs did not answer the question for vignette B (*n* = 67), three GPs did not answer the question for vignette C (*n* = 31), two GPs did not answer the question for vignette D (*n* = 54), E (*n* = 29) and F (*n* = 53)

Figure 4 shows the subsequent actions of respondents whose initial action was to perform a 12-lead ECG, given the results were negative for AF. In all cases, the majority chose to continue the diagnostic process. GPs would refer patients to a cardiologist more often in case of signs of AF during physical examination (vignette B, D and F; *n* = 13, 13 and 11), than when patients did not show any signs (vignette A, C and E; n = 1, 2 and 1).

GPs preferred short-term above long-term monitoring in all cases. In case of frequent symptoms of AF (vignette E, F), respectively 40 and 36% of GPs would choose short-term monitoring at any moment in the diagnostic process, and 19 and 11% would choose long-term monitoring. In case of non-frequent symptoms (vignette C, D) respectively 31 and 29% would choose short-term and 30 and 21% would choose long-term monitoring. Symptom frequency and the chosen monitoring duration were negatively associated in vignette C and vignette E (*p* = 0.031); i.e. GPs chose long-term monitoring 1.5 times more often in case of non-frequent symptoms than in case of frequent symptoms, and they chose short-term monitoring 1.3 times more often in case of frequent symptoms than in case of non-frequent symptoms. This association was not statistically significant for vignette D and vignette F (*p* = 0.125). In case of non-frequent symptoms, some GPs opted to instruct the patient to go for an ECG when the complaints occur.

### Actions after diagnosis

Almost all GPs would apply echocardiography (52/71, 73%). Thirty-seven percent (26/71) would refer the patient to a cardiologist who would then become the most responsible physician. Twenty-one percent of GPs (15/71) would refer the patient to a diagnostic centre, and 15% (11/71) would refer to a cardiologist only to perform echocardiography. Five GPs did not answer this question.

After diagnosing AF, 83% of GPs (59/71) would consider treating a patient themselves and 17% (12/71) would not. Often mentioned factors in this decision were patients age (41/71), the extent of complaints of AF (15/71), comorbidity (10/71), and other cardiac diseases (10/71). Five GPs did not answer this question. We found no significant association between years of experience and considering to start treatment themselves (*p* = 0.095).

### Comparison with guideline

Monitoring duration was shorter than recommended by the guideline (see Table 3 for details). The guideline does not recommend any form of screening for AF, the majority of GPs adheres to that advice by not applying any diagnostic tests in vignette A. If opportunistic screening in primary care proves to be effective, 83% (58/70) will support it. A 12-lead ECG is the first choice diagnostic test; most GPs follow this advice.

**Table 3:** Comparison of GPs responses to the vignettes with the Dutch guideline on AF diagnosis (*n* = 76)^a^

Case vignette	Guideline	Responding GPs (n)						
		12-lead ECG			Ambulatory monitoring			
		Yes	No^b^	Missing	Holter	Event recorder	None^c^	Missing
A: Only risk factors	No diagnostic tests	20	**53**	3	7	4	61	4
B: Signs	ECG	**71**	3	2	24	6	40	6
C: Non-frequent symptoms	ECG or event recorder. If negative ECG: event recorder	**34**	40	2	24	**26**	21	5
D: Signs & non-frequent symptoms	ECG or event recorder	**56**	17	3	24	**14**	33	5
E: Frequent symptoms	ECG or Holter. If negative ECG: Holter	**31**	42	3	**25**	19	27	5
F: Signs & frequent symptoms	ECG or Holter	**55**	18	3	**21**	13	37	5

^a^Bold numbers indicate the guidelines’ recommendation

^b^In this case ‘no’ means the GP did not choose to start the diagnostic process (for example would refer the patient to a cardiologist) or the GP would start the diagnostic process, but not with an ECG

^c^In this case ‘none’ means the GP did not start/continue the diagnostic process or did continue but chose something else, e.g. a repeated ECG measurement

## Discussion

In this study, GPs report that they are adequately equipped with devices, knowledge and experience to detect and diagnose AF. GPs adhere reasonably well to the guidelines in case vignettes concerning AF. Reported monitoring duration is often shorter than recommended. All GPs would undertake action in case a patient has signs or symptoms, and only a few would be satisfied if such a patient had a negative 12-lead ECG.

### Diagnostic equipment

In our study, 97% of GPs could order 12-lead ECGs, of whom 84% could do this in-house. These results are similar to the results of a study in the United Kingdom, which reported that all GPs had access to an ECG machine, of whom 81% (39/48) had an ECG device in their practice [14]. Taggar et al. identified access to the required equipment as a barrier for opportunistic screening, among others [14]. That does not match our findings, as Dutch GPs seem well equipped with diagnostic devices and our respondents did not mention that barrier. Taggar et al. did not further explore the current use of the diagnostic devices in practice. Our search revealed no additional articles on the availability of devices, nor on current practice of AF detection.

GPs judged that they have sufficient knowledge and experience to diagnose AF. Research by Compiet et al. shows that the diagnostic accuracy of GPs to detect AF is indeed high (96%) [15]. When comparing current results to our previous study among cardiologists, we see that monitoring devices are more often available to cardiologists than to GPs, as is to be expected [12]. Holter devices were available to 98% of cardiologists and 79% (60/76) of GPs, patient triggered event recorders were available to 77 and 71% (54/76), and automatically triggered event recorders to 42 and 22% (22/73), respectively.

### Diagnostic process

GPs chose shorter monitoring duration in case of frequent symptoms and vice versa. However, the chosen monitoring duration was still shorter than recommended in the guideline. In case of non-frequent symptoms, long-term monitoring is indicated, whereas more often short-term was chosen. Several studies show that short-term recording is not sufficient to diagnose paroxysmal arrhythmias in case of non-frequent symptoms [16, 17]. Our previous study showed that cardiologists also choose a shorter monitoring duration than recommended [12]. A possible reason for this is the assumed discomfort of long-term monitoring for patients. As shown in our current study, a lack of devices cannot explain this, as monitoring devices are readily available. Apart from innovating diagnostic methods and techniques, it might thus be worthwhile to optimise current care by extending the monitoring duration in order to improve AF detection. That might be a cheaper and less time-consuming way to improve AF detection rate than screening. Therefore, barriers to long-term monitoring should be identified and dealt with. Nevertheless, newer and less burdensome devices might be a solution [18].

### Strengths and limitations

To the best of our knowledge, this study is the first to explore the current practice of GPs regarding detection of AF. We compared the GPs’ reported actions with the current Dutch guideline. Two months after concluding our data collection, a revised version of this guideline appeared. We checked the two versions for differences in diagnostic recommendations and found none. Therefore, we consider our study results up-to-date. We did not compare GPs’ responses to other guidelines, because we wanted to compare them to the guideline they use in practice.

We asked GPs to assess the adequacy of their knowledge and experience regarding AF. Although GPs were very confident of their knowledge and skills, we need to be careful to draw firm conclusions, as self-assessment of competence by physicians is not necessarily accurate [19]. As compared to empirical studies using data from medical records, case vignette studies may have a lower validity regarding behaviour of GPs, but they are an efficient and well-accepted technique to explore choice behaviour and attitudes with a higher validity than regular questionnaires [20, 21].

Our study sample was small, but met our predefined sample size. Compared to the Dutch GP population (mean age 48 years, 49% male) [22]; our population was a little older (50.7 years) and counted more men (61.8%).

## Conclusion

Responding GPs stated to have adequate equipment, knowledge and experience to detect and diagnose AF. A 12-lead ECG is the preferred diagnostic tool by the majority of GPs, and most GPs can order or perform ECGs, without having to refer to a cardiologist. Duration of monitoring was often shorter than recommended by the Dutch guideline, suggesting that there may be room for improving the detection rate of AF by increasing the monitoring duration.

## Data Availability

The datasets used and/or analysed during the current study are available from the corresponding author on reasonable request.

## Abbreviations

AF: Atrial fibrillation
D_2_AF: Detecting and Diagnosing Atrial Fibrillation; study name
ECG: Electrocardiogram
GP: General practitioner
WMO: Medical Research Involving Human Subjects Act; in Dutch ‘Wet medisch-wetenschappelijk onderzoek met mensen’
