# Correction to: How do Dutch general practitioners detect and diagnose atrial fibrillation? Results of an online case vignette study

**DOI:** 10.1186/s12875-020-1097-2

**Published:** 2020-02-05

**Authors:** N. Verbiest-van Gurp, D. van Mil, H. A. M. van Kesteren, J. A. Knottnerus, H. E. J. H. Stoffers

**Affiliations:** 1 Care and Public Health Research Institute (CAPHRI), Department of Family Medicine, Maastricht University, Peter Debyeplein 1, 6229 HA Maastricht, Limburg The Netherlands; 2 Department of Cardiology, Admiraal de Ruyter Ziekenhuis, s-Gravenpolderseweg 114, 4462 RA Goes, Zeeland The Netherlands

**Correction to: BMC Fam Pract (2019) 20:175**

**https://doi.org/10.1186/s12875-019-1064-y**

Following publication of the original article [1], the authors opted to remove the authors full name from:

N. (Nicole) Verbiest-van Gurp^1^*, D. (Dominique) van Mil^1^, H.A.M. (Henri) van Kesteren^2^, J.A. (André) Knottnerus^1^, H.E.J.H. (Jelle) Stoffers^1^.

to

N. Verbiest-van Gurp^1^*, D. van Mil^1^, H.A.M. van Kesteren^2^, J.A. Knottnerus^1^, H.E.J.H. Stoffers^1^.

The original article has been corrected.
